# Blue toe syndrome caused by emboli from anomalous left atrial septal pouch thrombus: a case report

**DOI:** 10.1186/s12959-020-00226-x

**Published:** 2020-07-20

**Authors:** Snehasis Pradhan, Kciku Gresa, Jan-Peter Röing genannt Nölke, Hans-Joachim Trappe

**Affiliations:** Department of Cardiology and Angiology, Marien Hospital Herne, Ruhr- University of Bochum, Hoelkeskampring 40, 44625 Herne, Germany

**Keywords:** Blue toe syndrome (BTS), Left atrial septal pouch (LASP), Interatrial septum, Atrial fibrillation, CT angiography, Transesophageal echocardiography, Case report

## Abstract

**Background:**

Left atrial septal pouches (LASPs) are a relatively newly described but common anatomical cardiac variant thought to be associated with atrial fibrillation (AF) and cardio-embolic stroke. Blue toe syndrome (BTS) describes ischemic changes in the toes due to microembolisation of the digital arteries. Establishing the etiology of BTS is vital so that the underlying cause can be treated. Here we describe the first case of BTS arising due to emboli from LASP thrombus arising on a background of new-onset AF.

**Case presentation:**

A 65-year-old man presented with a two-day history of progressive painful swelling and bluish-purple discoloration of the second and fourth toes of his left foot and new-onset AF. Tests for hypercoagulability disorders were negative. Duplex ultrasound and CT angiography excluded deep venous thrombosis and an absence of embolus, thrombus, or occlusion in the arterial tree in the lower extremities bilaterally, so BTS was diagnosed. While transthoracic echocardiography and chest CT initially showed no cardiac abnormalities or mural thrombus, subsequent transesophageal echocardiography revealed a LASP with an associated pedunculated thrombus. The affected toes were amputated due to wet gangrene, but the patient recovered well with thrombus resolution after anticoagulation.

**Conclusion:**

The presence of a LASP in the absence of any other identifiable cause of BTS should trigger careful investigation of the interatrial septum, preferably using a multimodality imaging approach. The possibility that LASPs may not merely be an innocent bystander but a causative mechanism for peripheral ischemia must be considered.

## Background

“Blue toe syndrome” (BTS; also known as “trashfoot”) was first reported in 1976 and describes the sudden onset of acute pain and cyanosis in one or more toes due to microembolization of the digital arteries from a proximal source via a patent arterial tree in an otherwise well-perfused foot [1]. In the original case series describing BTS [2], all 31 patients had direct angiographic evidence of a proximal source of emboli in the vascular tree. The etiologies of BTS can be divided into three main groups: (i) emboli from the cardiac and arterial system; (ii) acquired hypercoagulability disorders; and (iii) syndromes leading to peripheral vascular pathology (Table 1) [1]. These causes are not mutually exclusive; for example, cryoglobulinemia can induce vasculitis and subsequent digital arteriolar and capillary thrombosis. Therefore, definitive subclassification of BTS can be difficult in some cases.

**Table 1:** Differential diagnosis of blue toe syndrome

Emboli from cardiac and arterial system	Hypercoagulability disorders	Peripheral vascular pathology
Left atrial septal pouch Atrial fibrillation Left ventricular aneurysm Cholesterol crystal Penetrating ulcers or Aneurysms of the aorto-iliac-femoral arterial system Cardiac/vascular tumors (myxoma) Valvular heart disease Endocarditis Iatrogenic injury	Antiphospholipid syndrome Malignancy Thrombocytopenic purpura Disseminated intravascular coagulation Warfarin skin necrosis Cryoglobulinemia Myeloproliferative disorders	Raynaud’s phenomenon Perniosis Frostbite Infectious and noninfectious inflammation Medication-induced vasoconstriction

In clinical practice, obliteration of the digital arterial vascular network can be devastating. In most instances, the cessation of arterial blood flow and consequent ischemic necrosis requires amputation of the affected toes or foot and may be life-threatening. Arterial embolisms are a primary cause of acute lower limb ischemia, with the majority of emboli, particularly in BTS, originating from the left heart as a result of intracavitary thrombus fragmentation [3]. Atrial fibrillation, left ventricular aneurysm, bacterial endocarditis, valvular heart disease, vascular tumors, and penetrating ulcerated atherosclerotic plaques in the aorto-iliac-femoral arterial system with or without aneurysm are the major sources of microembolisation [4]. A minority are paradoxical venous emboli that pass through an intracardiac shunt [5].

Atrial septal pouches are a relatively newly described but common anatomical cardiac variant [6]. An atrial septal pouch is a kangaroo pocket-like structure in the interatrial septum, without an interatrial shunt. They develop from postnatal partial fusion of the septum primum and the septum secundum, either opening into the left atrium or, less frequently, into the right atrium. Left atrial septal pouch (LASP) is very common, with an overall prevalence of 40.8%; right septal pouches and double pouches are present in 5.1 and 3.7% of individuals, respectively [7]. Although right septal pouches seem to have no clinical significance, the high prevalence of LASPs and evidence of a possible association between LASP and various cardio-embolic consequences, especially atrial fibrillation and cardio-embolic stroke [8], suggest that LASP may not be entirely benign. Therefore, LASP could also be a source of cardio-embolism in BTS, although this phenomenon has yet to be described in the literature.

Here we report the first case of thrombus formation within a LASP in the absence of a patent foramen ovale giving rise to BTS. This case illustrates the previously unrecognized potential of LASP thrombus giving rise to BTS, which in this case was complicated by digital necrosis, gangrene, and the eventual need for toe amputation.

## Case presentation

**Fig. 1:**
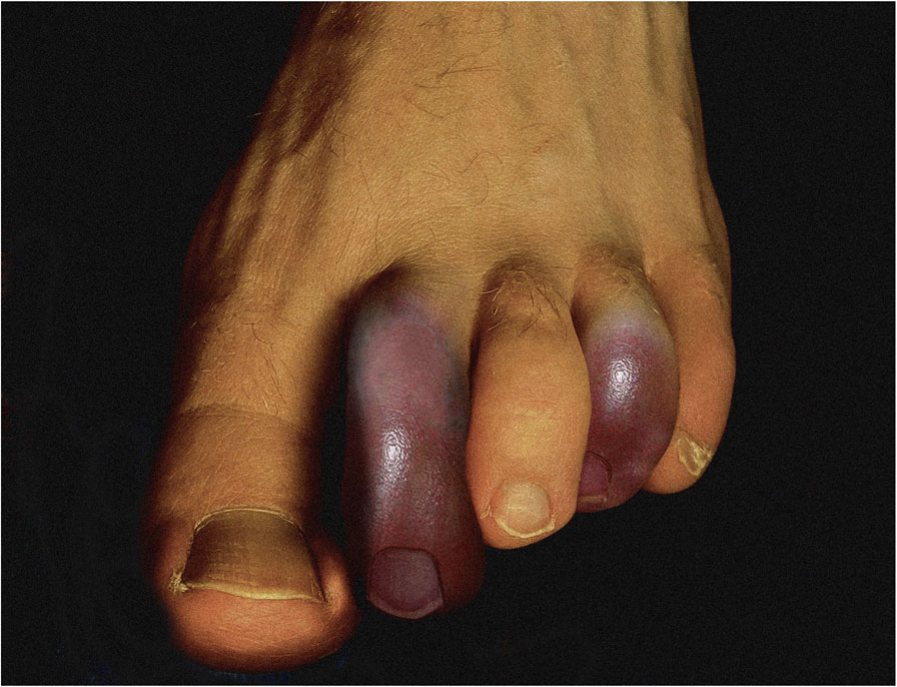
Bluish purple ischemic discoloration of the second and fourth toes of the left foot consistent with blue toe syndrome

A 65-year-old man presented to the emergency department with two-day history of progressive painful swelling and bluish-purple discoloration of his second and fourth toes of his left foot (Fig. 1) and history of palpitations for a week. There was no recent history of any trauma, fever, or excessive sensitivity or exposure to the cold. He had a past medical history of hypertension, for which he was prescribed ramipril, metoprolol, and amlodipine, and he was overweight.

On arrival, he was tachycardic at 188 bpm and his blood pressure was 110/70 mmHg. Clinical examination of his extremities revealed that the left second and fourth toes were tender, slightly edematous, and purple-blue, with a sharp demarcation between discolored and normal skin at the base of both digits. There were no signs of livedo reticularis or superficial lymphangitic streaking in the feet or lower extremities. BTS was suspected because peripheral pulses were palpable in both legs and there was no clinical evidence of ischemia in the contralateral limb.

His CRP was elevated at 10 mg/L (normal < 0.5 mg/L) and D-dimers were positive. Brain natriuretic peptide was elevated, but renal and liver function parameters were well within normal limits. Diagnostic screening for diabetes was negative. Laboratory tests for thrombophilia, which included disorders of plasminogen activation, antithrombin III deficiency, protein C and protein S deficiency, and serum homocysteine levels, were negative.

Left foot radiographs showed mild swelling but no evidence of osteomyelitis or fracture. Duplex ultrasound excluded deep venous thrombosis and confirmed patency of the outflow vessels without any embolus, thrombus, or occlusion in the arterial tree in the lower extremities bilaterally. A bedside transthoracic echocardiography (TTE) showed normal left ventricular systolic function without any segmental wall motion abnormality. The interatrial septum was thickened but there was no visible mobile echogenic mass in the atria. Signs of pulmonary hypertension were absent. An electrocardiogram showed tachycardia with new-onset irregularly irregular atrial fibrillation.

**Fig. 2:**
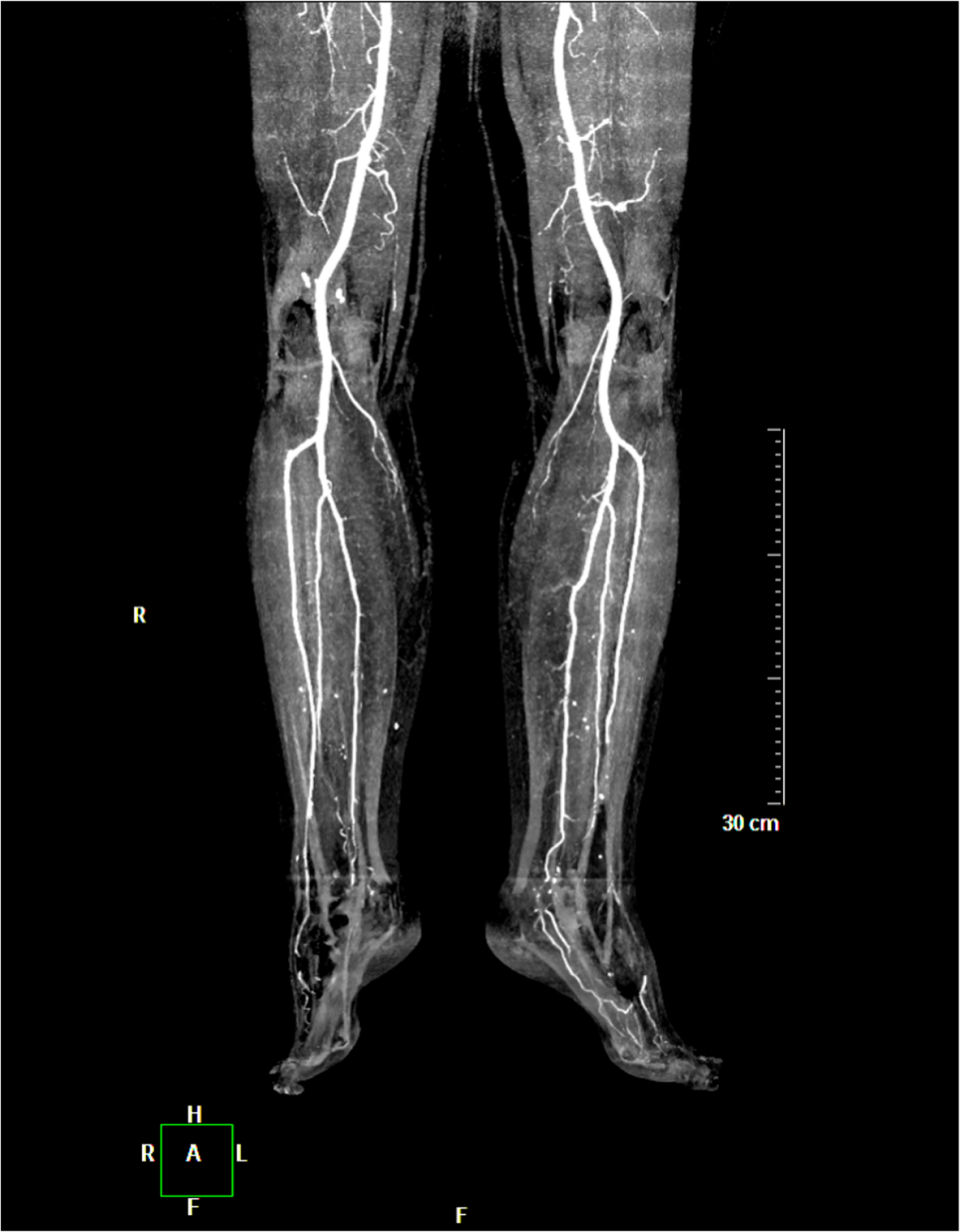
Coronal maximum intensity projection (MIP) of lower extremity CT angiography ruling out any significant arterial stenosis

**Fig. 3:**
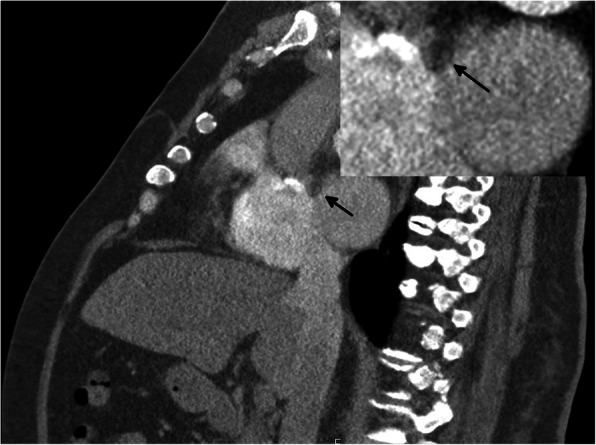
Chest computed tomographic image demonstrating a filling defect adjacent to the interatrial septum (arrow)

The patient underwent immediate thoracic, abdominal, and lower limb contrast-enhanced computed tomographic angiography (CTA), which excluded any proximal thromboembolic source or occlusions in the arterial tree (Fig. 2). There was no evidence of abdominal aortic aneurysm or severe atheromatous plaque. Chest CT incidentally revealed a focal left atrial cavity filling defect adjacent to the atrial septum. This defect was initially suspected to be a cardiac CT artifact in light of the irregular tachyarrhythmia at the time of acquisition, which can generate slow-moving, turbulent blood due to atrial hypokinesia (Fig. 3).

Initially, it was decided to treat the case conservatively with anticoagulant (heparin drip) and anti-aggregate medications (intravenous PGE1). He was admitted to vascular surgery with a diagnosis of acute limb ischemia. He was placed on an empiric antibiotic regimen (ciprofloxacin), received morphine for pain, and was sent to the telemetry unit.

However, the patient experienced extreme worsening of progressive pain overnight that prevented sleep and further bluish discoloration of both toes. Since he had palpable posterior tibial and dorsalis pedis pulses and a normal ankle-brachial index (1.2; normal > 1), the vascular surgery team decided against toe amputation. An emergency percutaneous vascular angiography showed no further evidence of any major arterial occlusion of the arterial tree of the left limb.

**Fig. 4:**
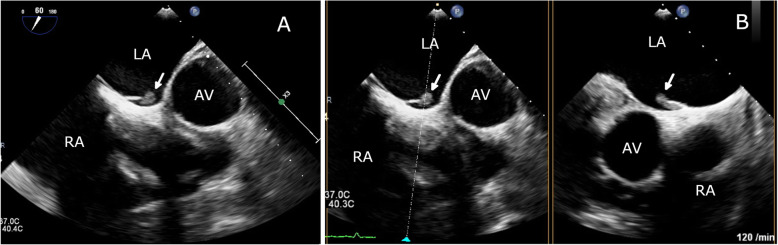
Transesophageal echocardiogram (TEE) showing LASP opening into the left atrial (LA) cavity and a 7.7 × 3 mm thrombus (arrow), with the site of attachment corresponding to the LASP (2-dimensional TEE images at 60° [**a**] and biplane TEE image [**b**] of the interatrial septum). AV, aortic valve; LA, left atrium; RA, right atrium

**Fig. 5:**
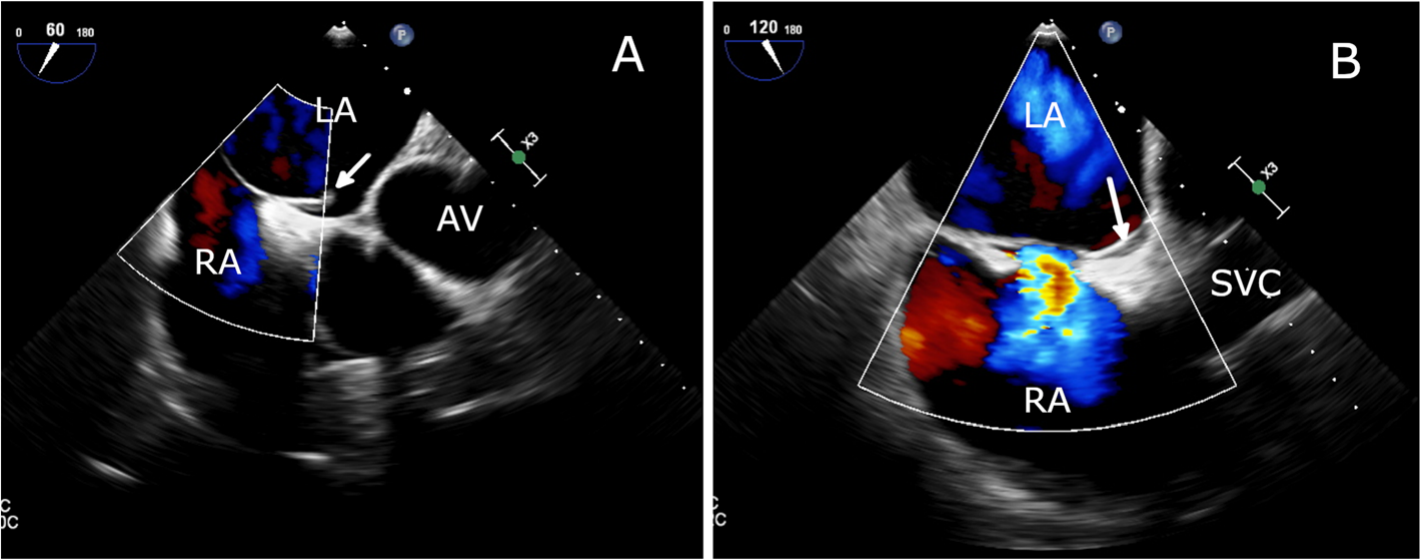
Left atrial septal pouch thrombus seen by color Doppler imaging during TEE at 60° [**a**] and 120° [**b**] and simultaneously excluding any probable atrial septal defect

**Fig. 6:**
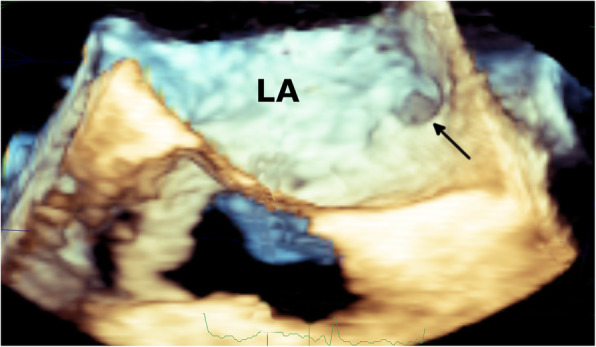
3D reconstruction of the TEE image of the left side of the interatrial septum to visualize the sessile thrombus (arrows) adherent to the septum and arising from the left atrial septal pouch


Given the high index of suspicion of an intracavitary thrombus in the setting of new-onset AF and progression of the cyanotic toes, the patient was referred to cardiology, where transesophageal echocardiography (TEE) revealed an anomalous LASP and a 7.7 × 3 mm mobile ovoid echodense mass with a narrow stalk attached to the pouch, suggestive of thrombus (Fig. 4**,** Supplemental Video 1). There was no evidence of an atrial septal defect on color Doppler flow imaging (Fig. 5), although it showed mild mitral regurgitation. A further confirmatory agitated saline test to exclude atrial septal defect was not performed to avoid dislodging the thrombus. No mass was seen in the left atrial appendage (LAA; peak emptying velocity 63 cm/s). Three-dimensional (3D) TEE visualized the LASP containing a mobile thrombus with attachment of its stalk to the interatrial septum (Fig. 6 and Supplemental Video 2). It is noteworthy that the exact shape and volume of the mass were revealed by the 3D acquisition.

**Additional file 1**: **Video Supplement 1.** Biplane TEE image of the interatrial septum showing LASP opening into the left atrial cavity and a sessile thrombus.



**Additional file 2: Video supplement 2.** 3D reconstruction of the TEE image of the left side of the interatrial septum to visualize the sessile thrombus adherent to the septum and arising from the left atrial septal pouch.


The AF was managed with metoprolol, to which the patient responded adequately. Taking the small size of thrombus into consideration, absence of direct evidence of transit of thrombus in the right atrium, and following multidisciplinary case discussions involving cardiology, vascular surgery, and cardiac surgery, the decision was made to continue therapeutic anticoagulation and observe. Despite the intravenous administration of ciprofloxacin and later tazobactam, after microbiological wound swab culture of normal flora (*Staphylococcus aureus* and *Proteus species*), the patient developed wet gangrene and demarcated necrosis in his second and fourth toes. Antibiotic therapy was stopped given the lack of apparent benefit. The patient was offered amputation, to which he agreed due to the likelihood of faster healing and rehabilitation. Histopathological examination of the resected specimen confirmed tissue necrosis without signs of malignancy.

**Fig. 7:**
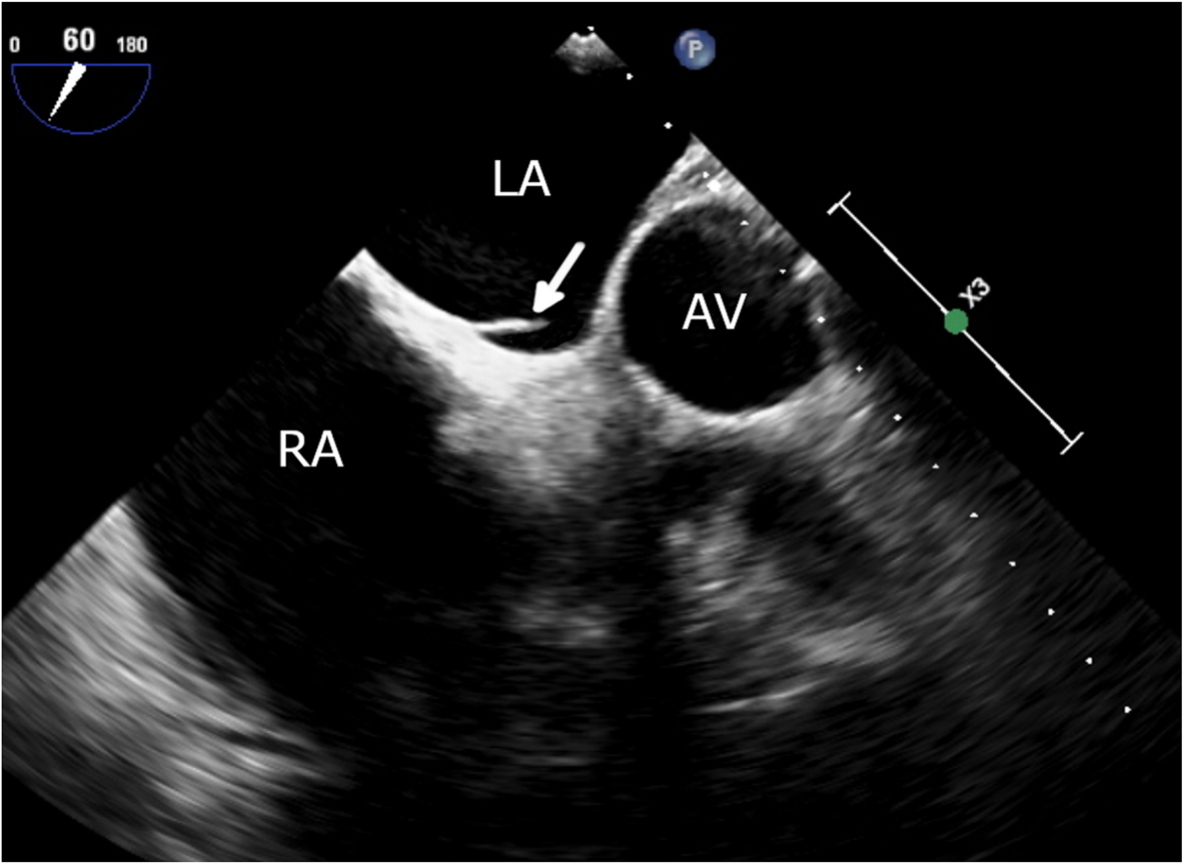
Follow-up TEE showing LASP opening into the left atrium with complete resolution of thrombus after 3 months of oral anticoagulation

**Fig. 8:**
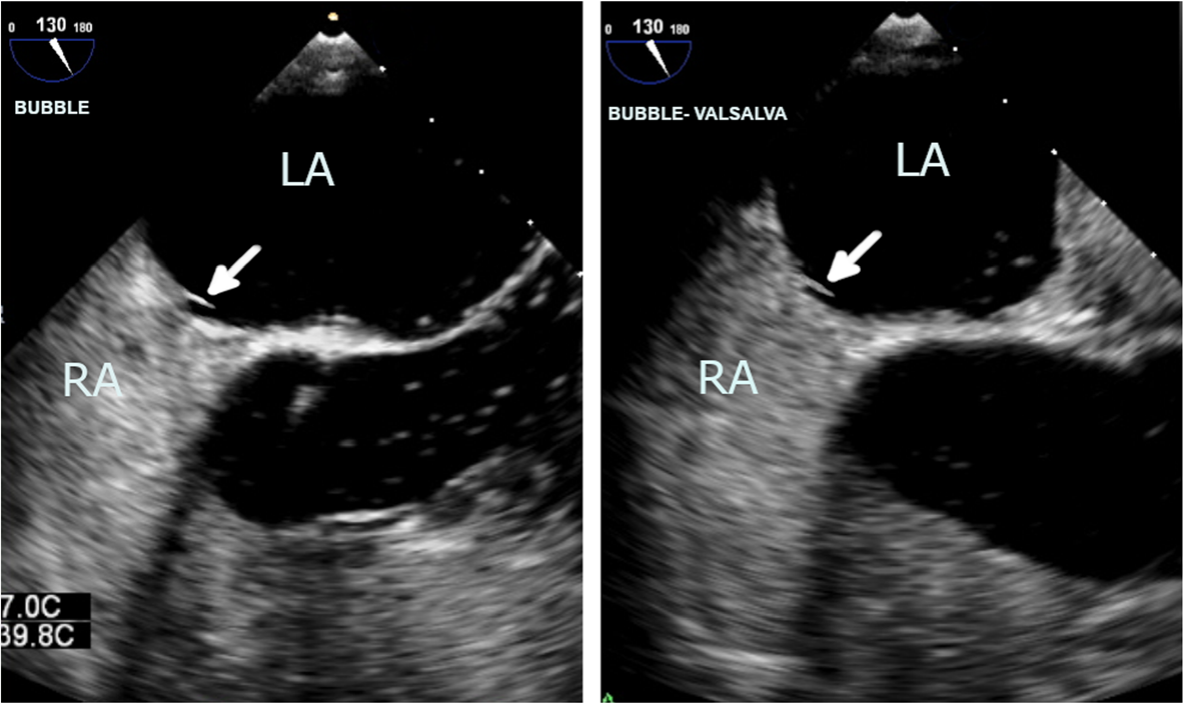
Agitated saline contrast imaging in a follow-up TEE with the bicaval view showing a LASP (arrow) and absence of an atrial septal defect both at rest (**a**) and with Valsalva maneuver (**b**)

His incisions healed 2 weeks later and, after a short period of physiotherapy, he was able to ambulate. No further infection was detected. The patient was transitioned to an oral anticoagulant (apixaban) and he was discharged from the hospital. He remained well 3 months after discharge, with follow-up TEE showing no evidence of thrombus (Fig. 7). On further imaging with agitated saline contrast, there was no evidence of an atrial septal defect (Fig. 8).

Extensive tests for autoimmune disorders potentially responsible for thrombotic events, including anti-lupus erythematosus, antinuclear, antimitochondrial, and antiphospholipid antibodies, were all negative. Therefore, the LASP, together with new-onset AF, were likely to have resulted in thrombus formation within the septal pouch with subsequent embolization to the digital artery of the left foot to cause BTS.

## Discussion and conclusions

Here we describe a unique case of LASP thrombosis causing catastrophic BTS necessitating toe amputation. Careful history taking and physical examination will often suggest the etiology of BTS, but in this case, the etiology was more difficult to establish. The spontaneous onset of painful bluish discoloration of the toes in BTS usually signifies an arterial embolic disorder, and emboli can be dislodged thrombi, fragments of atheromatous plaque, or rarely, tumor cells or other foreign bodies [4, 9]. Therefore, thrombogenic and atherogenic sources of emboli need to be considered in the diagnostic work-up. Establishing the underlying cause of BTS is important since, if untreated, BTS arising from embolization can frequently be complicated by further emboli that not only result in the loss of digits but also the forefoot and limb, sometimes causing death [10].

In our case, the patient presented with acute onset painful and cyanotic second and fourth toes of the left foot. A thrombogenic etiology initially seemed more likely in this patient given the negative history of trauma, new-onset tachycardia and AF, and distal ischemia appearing simultaneously in multiple toes. Non-invasive vascular assessment such as Duplex ultrasound (DUS) is the first-line imaging modality in any form of acute limb ischemia (ALI) due to its diagnostic accuracy, wide availability, low cost, rapidity, and non-invasiveness. DUS can accurately determine the presence of an aneurysm and the anatomic location and degree of thromboembolic obstruction, hence should be performed in all patients [11]. Neither aneurysm nor thromboembolic obstruction were apparent on DUS of the arteries proximal to the ischemic digits, contralateral arteries, and the venous system in our patient at the time of presentation. Alternatively, CT angiography (CTA) is a rapid and accurate imaging modality that can diagnose and grade the extent of vascular disease in ALI and is the preferred imaging modality for establishing the source of the embolism [12]. In our patient, CTA showed no evidence of abdominal aortic aneurysm, atheromatous plaque, or any source of emboli proximal to the digital ischemia. Percutaneous vascular angiography, while considered the “gold standard” test [13], is mostly reserved for cases in which non-invasive evaluation is uninformative and in our case was inconclusive for any immediate thromboembolic event. Of note, invasive angiography should not be used as the first diagnostic modality and should not replace non-invasive imaging for the definitive diagnosis of BTS.

TTE is a useful screening tool for detecting a potential cardiac source of peripheral embolism [14]. In our patient, TTE revealed no visible signs of ventricular aneurysm, vegetations, intracavitary masses, or thrombus. A failure to visualize the source of an embolus on TTE does not rule out its existence, so routine TTE can be omitted when there is a clear cardiac source of embolism clinically.

Other possible differential diagnoses of the etiology of toe ischemia included hyperviscosity disorders such as cryoglobulinemia, antiphospholipid syndrome, thrombotic thrombocytopenic purpura, and disseminated intravascular coagulation [15]. Cryoglobulinemia may cause purpuric or gangrenous lesions in the distal extremities, and its incidence is increasing due to its association with hepatitis C infection [16]. However, in cryoglobulinemia, skin lesions would typically be more widespread and unlikely to affect, as in our case, only two toes. Antiphospholipid syndrome is an autoimmune disease causing thrombotic events in both the arterial and venous systems. Thrombotic thrombocytopenic purpura causes BTS through occlusion of arterioles with platelet plugs. Disseminated intravascular coagulation typically arises secondary to another condition such as sepsis or malignancy, causing widespread activation of the clotting cascade generating excess thrombin and resulting in vascular occlusion. Extensive laboratory and clinical investigations for these conditions were all normal or negative in our case.

Several vasoconstrictive disorders such as Raynaud’s phenomenon, perniosis, and frostbite may cause BTS through exposure to cold or even emotional stress [17]. In perniosis, digital cyanosis results from exposure to low temperatures (above freezing) and damp and is often accompanied by blisters, erosions, and eventually ulcers. In BTS secondary to vasculitides, inflammatory white blood cells adhere to and damage vessel walls, leading to narrowing, occlusion, and possible rupture of the small- and medium-sized vessels. Reynaud’s phenomenon was unlikely in our patient given the limited involvement of two toes without any visible cyanosis in the upper extremities. Vasculitis was improbable, considering the sharply demarcated border between the affected and unaffected skin, the absence of fever, and lack of response to antibiotic therapy. Taking the negative history of cold exposure into consideration, perniosis or frostbite were unlikely etiologies.

Many routine medications and recreational drugs such as cocaine and cannabis have been reported to precipitate BTS. Prescribed medications reported to cause BTS include systemic vasopressors (epinephrine, dopamine), imipramine, amphotericin B, and warfarin, which may trigger transient deficiency of protein C [18]. Physicians should therefore be aware of the side effects of these drugs and medications. In our case, the patient’s prescribed medications had no such thrombogenic potential.

TEE identified a LASP with an ovoid thrombus projecting into the left atrium with its peduncle entrapped in the septal pouch. No other embolic source was identified. Moreover, the possibility of a septic emboli in the absence of endocardial infect focus was rather unlikely. Of note, infective endocarditis is the most common embolic source in septic emboli.

A 3D TEE provided further comprehensive evaluation of the interatrial septum with further confirmation of the presence of thrombus. 2D and 3D TEE are superior to TTE for identifying anatomic anomalies of the interatrial septum, since TEE may not adequately visualize the anatomy [19]. Atrial septal pouches can also be identified by CT imaging, and physician must be able to recognize it. However, CT is not required in patients who have already undergone TEE and is not the diagnostic modality of choice. Indeed, in our case, the LASP was initially considered to be an artifact on CT, with a later comparison of the filling defect adjacent to the atrial septum in CT images with TEE evaluation of the thrombus showing an identical morphology and securing the diagnosis. TEE seems to be superior to CT for the clinical identification and comprehensive evaluation of atrial septal pouches. Moreover, TEE should be the preferred imaging technique due to its ability to unambiguously detect patent foramen ovale.

The high prevalence of atrial septal pouches (over 50% of the healthy population), especially LASPs in ~ 40% of healthy individuals, means that the pouch is not a pathology rather a normal human anatomical variant (7). LASPs can promote blood stasis and are a potential site for thrombus formation according to Virchow’s triad. The most frequent cause of atrial thrombus formation is AF, which generally occurs in mitral valve lesions of rheumatic origin, hyperthyroidism, acute myocardial infarction, and cardiosclerosis. It has been suggested that LASPs are associated with a higher prevalence of AF, perhaps as a consequence of the developmental fusion of the PFO or perhaps related to the presence of scar tissue in the septal pouch apex, which may be proarrhythmogenic [20]. The hemodynamic changes caused by AF, causing the formation of mural thrombi within LASPs, are also a critical source of emboli. Apart from AF, several other reported risk factors for pouch thrombogenesis include elevated left ventricular filling pressure in heart failure, possible local endothelial injury, and a hypercoagulable state.

The relevant discussion point in our case was to establish the relationship between LASP, AF, and BTS. In reality, it is difficult to know whether the AF precipitated the thrombus, whether the LASP triggered the AF, or whether the thrombus formed in the LASP and the new-onset AF was incidental - but the important educational point is that these were the undeniable possibilities and LASPs have been reported in association with AF, transient ischemic attacks and thrombi along the left atrial septum [6–8]. However, there is much to be learned before one can place such a discovery in perspective.

Through multimodal imaging, our case confirmed thrombus formation inside a LASP in the setting of new-onset AF. In non-valvular AF, left atrial thrombi almost exclusively arise in the LAA due to decreased velocity (< 20–30 cm/s). However, in our case, no thrombi were detected in the LAA. Taking this into account and extensive exclusion of other possible etiologies, we suggest that the LASP was the most likely source of emboli causing lower extremity digital ischemia.

As noted above, numerous conditions can lead to toe necrosis, so management is entirely dependent on the cause. Although we had no definite etiology at the time of establishing treatment, we took appropriate and safe action to minimize ischemic insult and prevent progression by prescribing anticoagulation and a vasodilator (PGE1). In the clinical setting of critical ischemia of uncertain etiology, a cocktail protocol including anticoagulation, vasodilators, antiplatelet agents, and eventually pulsed glucocorticoids with or without vasodilatory prostaglandins (PG) should be considered as a salvage treatment until a definite diagnosis is made. Dry gangrene is not an emergency and can be treated conservatively before any final surgical intervention. However, wet gangrene is a semi-emergency because it may cause cellulitis, sepsis, and severe necrosis, and it often warrants immediate surgical attention. For better functional outcomes and faster rehabilitation, as in our case, amputation may be the best approach.

To conclude, BTS is an acute limb ischemia that is frequently misdiagnosed on initial presentation. Since pedal pulses are often palpable, the physician may discount vascular pathology. Our case demonstrates that LASPs may serve as a nidus for thrombus formation, particularly in patients with impaired cardiac function and stagnant atrial flow. LASPs can therefore be a source of embolic complications leading to BTS. We suggest that the presence of a LASP in the absence of any other risk factor should trigger careful investigation of the interatrial septum, preferably using a multimodality imaging approach for the diagnosis of LASP masses. The possibility that LASPs may not simply be an innocent bystander but a causative mechanism for peripheral ischemia must be considered.

## Abbreviations

AF: Atrial fibrillation
ALI: Acute limb ischemia
BTS: Blue toe syndrome
CRP: C-reactive protein
CTA: Computed tomography angiography
DUS: Duplex ultrasound
LAA: Left atrial appendage
LASP: left atrial septal pouch
PGE: Prostaglandin E
TEE: Transesophageal echocardiography
TTE: Transthoracic echocardiography

## References

[1] Blue Toe Syndrome. https://www.escardio.org/Journals/E-Journal-of-Cardiology-Practice/Volume-2/Blue-Toe-Syndrome-Title-Blue-Toe-Syndrome. Accessed 24 Mar 2020.

[2] Karmody AM (1976). “Blue toe” syndrome: an indication for limb salvage Surgery. Arch Surg.

[3] Becquemin J-P, Kovarsky S (1995). Arterial emboli of the lower limbs: analysis of risk factors for mortality and amputation. Ann Vasc Surg.

[4] Making the diagnosis when the patient has “blue toes”. - PubMed - NCBI. https://www.ncbi.nlm.nih.gov/pubmed/7982584. Accessed 30 Mar 2020.

[5] Greenberg JW, Goff ZD, Mooser AC, Wittgen CM, Smeds MR. Acute Limb Ischemia Secondary to Patent Foramen Ovale–Mediated Paradoxical Embolism: A Case Report and Systematic Review of the Literature. Ann Vasc Surg. 2020;:S0890509619310659. 10.1016/j.avsg.2019.12.022 31904517

[6] Krishnan SC, Salazar M (2010). Septal pouch in the left atrium. JACC Cardiovasc Interv.

[7] Mazur M, Jasinska KA, Walocha JA (2018). The morphology, clinical significance and imaging methods of the atrial septal pouch: a critical review. Transl Res Anat.

[8] Gurudevan SV, Shah H, Tolstrup K, Siegel R, Krishnan SC (2010). Septal Thrombus in the left atrium: is the left atrial Septal pouch the culprit?. JACC Cardiovasc Imaging.

[9] Sidawy AN, Perler BA (2018). Rutherford’s Vascular Surgery and Endovascular Therapy.

[10] Brewer ML, Kinnison ML, Perler BA, White RI (1988). Blue toe syndrome: treatment with anticoagulants and delayed percutaneous transluminal angioplasty. Radiology..

[11] Olinic D-M, Stanek A, Tătaru D-A, Homorodean C, Olinic M. Acute limb ischemia: an update on diagnosis and management. J Clin Med. 2019;8. 10.3390/jcm8081215. 10.3390/jcm8081215 PMC6723825 31416204

[12] Oweis Y, Viets Z, Shetty AS (2017). Role of lower extremity run-off CT angiography in the evaluation of acute vascular disease. Abdom Radiol.

[13] Singh H, Cardella JF, Cole PE, Grassi CJ, McCowan TC, Swan TL (2002). Quality improvement guidelines for diagnostic arteriography. J Vasc Interv Radiol.

[14] Gossage JA, Ali T, Chambers J, Burnand KG (2006). Peripheral arterial embolism: prevalence, outcome, and the role of echocardiography in management. Vasc Endovasc Surg.

[15] Hirschmann JV, Raugi GJ (2009). Blue (or purple) toe syndrome. J Am Acad Dermatol.

[16] Hyperviscosity Syndrome in Cryoglobulinemia: Clinical Aspects and Therapeutic Considerations. Semin Thromb Hemost. 2003;29:473–8. 10.1055/s-2003-44555 14631547

[17] Brown PJ, Zirwas MJ, English JC (2010). The purple digit: an algorithmic approach to diagnosis. Am J Clin Dermatol.

[18] Aboud AA, Abrams M, Mancini AJ (2011). Blue toes after stimulant therapy for pediatric attention deficit hyperactivity disorder. J Am Acad Dermatol.

[19] Zisa D, Faletra FF, Wessler BS, Halin NJ, Reddy P, Patel AR (2019). Ridges and pouches: a case series of anomalous atrial Septal fusion. CASE Cardiovasc Imaging Case Rep.

[20] Hołda MK, Koziej M, Wszołek K (2017). Left atrial accessory appendages, diverticula, and left-sided septal pouch in multi-slice computed tomography. Association with atrial fibrillation and cerebrovascular accidents. Int. J. Cardiol.

